# Why should I use social chatbots? On potential users’ acceptance and the role of anthropomorphism

**DOI:** 10.3389/fpsyg.2026.1656749

**Published:** 2026-02-17

**Authors:** Marco Rüth, Justus M. Eifler, Anna Celina Schneider

**Affiliations:** Department of Psychology, University of Cologne, Cologne, Germany

**Keywords:** anthropomorphism, artificial intelligence, attachment, social chatbots, social support, technology acceptance, trust, usage intention

## Abstract

**Introduction:**

Chatbots can provide task-related services but also act as empathic conversation partners allowing for social interactions. Focusing on such social chatbots, we considered several theoretical frameworks to investigate potential users’ intention to use social chatbots and focused on the role of anthropomorphism.

**Method:**

Based on an online survey with 180 participants, we examined the role of 14 personal characteristics in potential users’ acceptance of social chatbots based on bivariate correlations and multiple regression analysis. Based on a subsequent within-subjects experiment and repeated measures analysis of variance, we also investigated differences in the intention of potential users to use more human-like versus less human-like social chatbots regarding their avatar and name.

**Results:**

Most personal characteristics were significantly correlated with participants’ intention to use social chatbots. The multiple regression model explained about 75% of variance in participants’ intention and identified experience and attitude regarding social chatbots as particularly important personal characteristics. Further, perceived usefulness, subjective norm, and perceived behavioral control regarding social chatbots as well as social support showed specifically strong bivariate correlations. The experimental part revealed that more human-like social chatbots received slightly yet significantly higher intention ratings.

**Discussion:**

We identified relevant personal characteristics for potential users’ intention to use social chatbots and found that potential users prefer using social chatbots with a more human-like appearance. While anthropomorphism can affect potential users’ intention to use social chatbots, other aspects seem more important. Overall, our findings provide valuable starting points to better understand why people intend to use social chatbots.

## 1 Introduction

Chatbots have become increasingly relevant in economic contexts but also in private settings (e.g., Maslej et al., 2024; Rapp et al., 2021). Chatbots are intelligent systems with a diverse range of applications, ranging from service encounters and health advisors to empathic conversation partners (e.g., Bilquise et al., 2022; Gopinath and Kasilingam, 2023). In the following, the term social chatbots refers to chatbots in their role as emotionally intelligent conversation partners who take an empathic rather than a task-directed role and who can provide companionship, emotional support, and entertainment to a broad audience (cf. Zhou et al., 2020). Examples of popular social chatbots include *Anima, Kajiwoto, Paradot*, and *Replika*. Previous studies have investigated several cognitive, social and emotional aspects of chatbots (see, e.g., Rapp et al., 2021). For instance, conversations with social chatbots can provide daily companionship and empathy (Ta et al., 2020), yet empathic messages were also found to cause irritation in novice users of chatbots (Urakami et al., 2019). Such irritation could be due to issues with distinguishing chatbots from real humans and the so-called *uncanny valley* (Mori et al., 2012), which can be related to negative feelings and reactions towards chatbots. Moreover, deficit-oriented approaches have criticized computer-mediated communication for lacking several qualities compared to real communication with humans, such as reduced sensory, socio-emotional, and contextual information. Still, complementary approaches such as the perspective of social information processing highlight that even text-based computer-mediated communication provides social cues and that immediate access and feedback can also contribute to social relationships (Winter et al., 2023). The complementary perspective is corroborated by a systematic literature review on interactions between humans and text-based chatbots, indicating that emotions play an important role and are sometimes even requested by users (Rapp et al., 2021). While the similarity between chatbots and humans (anthropomorphism) is related to user behavior and should be configured considering contextual factors, we focus on understanding potential users’ acceptance and the role of anthropomorphism regarding social chatbots.

**FIGURE 1:**
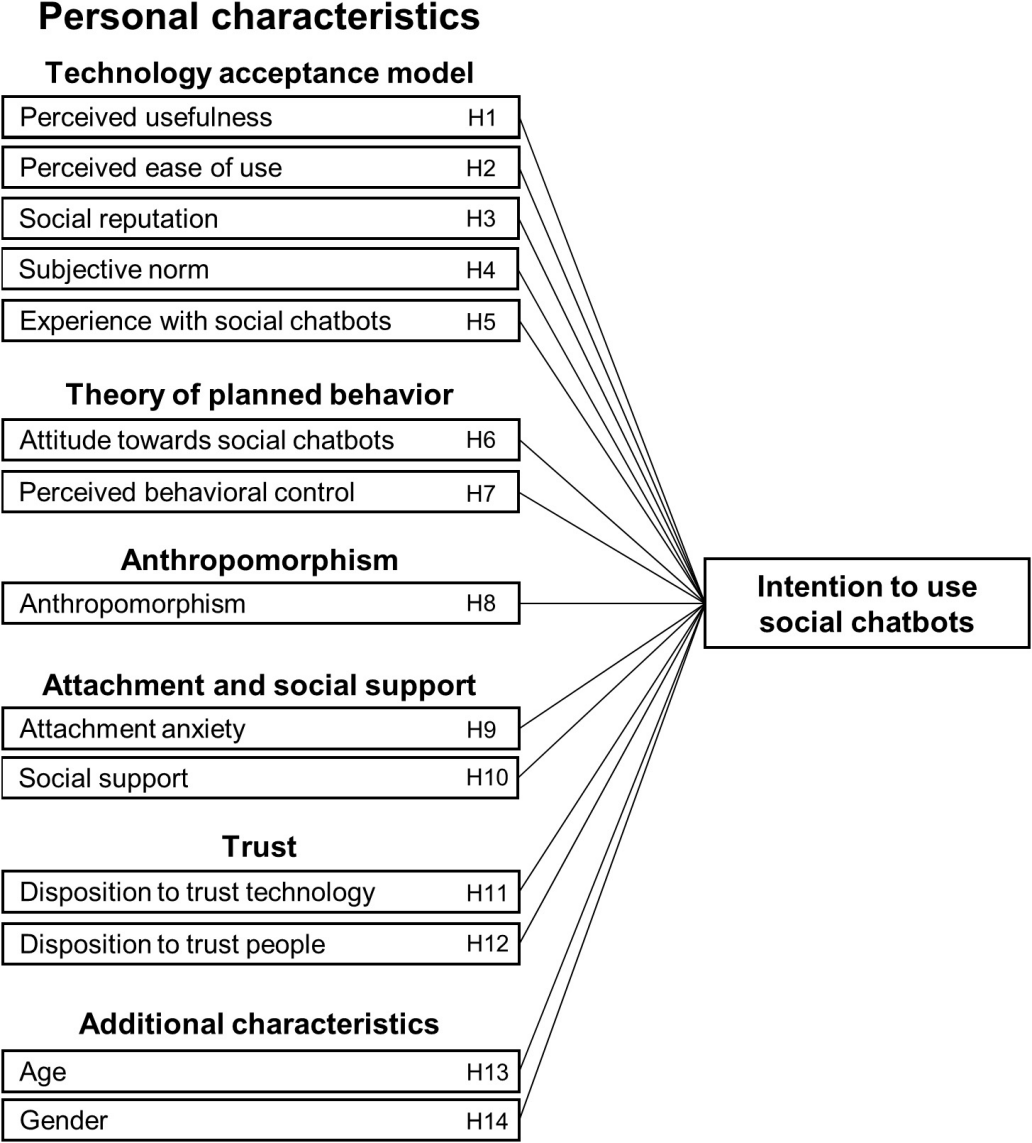
Research model on the role of personal characteristics in the intention to use social chatbots.

The acceptance of potential users—people who do not yet use social chatbots regularly in their daily life—can be understood in terms of their intention to use social chatbots, i.e., to exchange messages with a social chatbot for entertainment and social interaction during leisure time. For instance, a systematic literature review on AI-based conversational agents indicates that usage convenience, perceived usefulness, trust, enjoyment, and attitude towards technology are important aspects for the adoption of chatbots (Mariani et al., 2023). These findings are in line with a meta-analysis on the adoption of AI-based chatbots highlighting the relevance of attitude, perceived usefulness, and trust, while economic level and gender were identified as moderators (Li et al., 2023). Thus, several factors play a role in the intention to use chatbots and chatbot-like tools in general, but their relative importance and the intention to use social chatbots yet need to be examined. Hence, we scrutinize the role of several constructs in the usage intention of potential users of social chatbots. Figure 1 shows our research model that we elaborate on in the following.

### 1.1 Potential users’ acceptance of social chatbots

The technology acceptance model suggests that perceived usefulness and perceived ease of use are key predictors of intention and use of technologies (Venkatesh and Bala, 2008). Regarding the use of social chatbots, perceived usefulness refers to the subjectively experienced probability that social chatbot usage could improve the extent to which the goals of entertainment and social interaction are fulfilled. Prior research on chatbots found that perceived usefulness is positively related to the intention to use chatbots in service and educational contexts (Al-Abdullatif, 2023; Alotaibi and Hidayat-ur-Rehman, 2025) as well as AI-based chatbots (Li et al., 2023). Perceived ease of use means how effortless using a technology is and may vary due to the functional complexity of the type of chatbot under investigation (e.g., Al-Abdullatif, 2023; Mariani et al., 2023; Wang et al., 2024). The technology acceptance model further suggests that usage intention is determined by the extent to which using a technology is expected to enhance social reputation, to what extent significant others think a behavior should be exhibited (subjective norm), and by the extent of prior experience with a technology (Venkatesh and Bala, 2008). Subjective norm was found to be a relevant factor for usage intention, particularly for chatbot use in non-transactional contexts like leisure activities or information seeking (Gopinath and Kasilingam, 2023). Against this background, we expected positive relationships between participants’ intention to use social chatbots and perceived usefulness (H1), perceived ease of use (H2), the social reputation of using social chatbots (H3), subjective norm (H4), and experience with social chatbots (H5).

According to the theory of planned behavior (Ajzen, 2020), usage intention is also determined by how beneficial or detrimental one evaluates a behavior (attitude) and how easy one considers realizing a behavior (perceived behavioral control) (Ajzen, 1991). Previous research on chatbots in general suggests that attitude is a relevant factor for usage intention (De Cicco et al., 2020; Mariani et al., 2023). Thus, based on the theory of planned behavior and previous findings, we expected positive relationships between participants’ intention to use social chatbots and their attitude (H6) and perceived behavioral control (H7) regarding social chatbots.

Anthropomorphism theory postulates that people more likely assign human traits to non-human entities when information related to humans is available (Epley et al., 2007). In this regard, chatbots may have specific visual, interactional, and functional features and, thus, provide different anthropomorphic cues regarding, e.g., visual representation, demographic information, and verbal and nonverbal communication (Seeger et al., 2021; Xin and Liu, 2025; Zhang et al., 2025). In line with anthropomorphism theory, meta-analytic results suggest that the more human-like service chatbots are, the more people intend to use them (Gopinath and Kasilingam, 2023). Here, we therefore expected a positive relationship between participants’ intention to use social chatbots and the perceived anthropomorphism of social chatbots (H8).

Attachment theory highlights the importance of relationships with others across the lifespan for development and wellbeing (Bowlby, 1969/1991; Scharfe, 2021). Prior research indicates that social chatbots are considered by users as reference persons (Pentina et al., 2023; Xie et al., 2023). Anxious attachment seems to be particularly relevant as users of social chatbots were found to experience a salient level of anxiety about losing their social chatbot (Skjuve et al., 2022), despite a low probability to be rejected by a social chatbot due to their availability and affirmative nature (Xie et al., 2023). Here, we expected a positive relationship between participants’ intention to use social chatbots and their level of anxious attachment (H9).

While anxious attachment deals with the fear of losing social contacts, social support describes the existence and quality of social contacts (Vonneilich and Franzkowiak, 2022). Social support can be understood in terms of sources of support (e.g., family, friends), types of support (e.g., task-oriented, emotional), and quantity and quality of support (e.g., availability, adequacy) (Lin et al., 2018). Particularly in the context of the Covid-19 pandemic, people who experienced low social support were found to show a higher tendency to use social chatbots (Pentina et al., 2023). Accordingly, we expected a negative relationship between participants’ intention to use social chatbots and their level of social support (H10).

Another important factor for the intention to use technology is the trust in technologies such as chatbots (Gopinath and Kasilingam, 2023). In general, trust means the willingness to be vulnerable to the actions of someone else for relevant matters independently of their ability to monitor or control those actions (Mayer et al., 1995). It was found that trust in a chatbot used for recruitment purposes was crucial for the acceptance of the chatbot (Akram et al., 2024). Particularly regarding social chatbots, trust in human beings could also play a role in their acceptance. For instance, it was found that especially people with few social contacts form relationships with social chatbots (Pentina et al., 2023). Hence, we expected that participants’ intention to use social chatbots is positively related to their trust in technologies (H11) and negatively related to their trust in other people (H12).

Finally, the intention to use social chatbots may be different regarding age and gender. For instance, a stronger affinity for using social chatbots was found in younger (Chattaraman et al., 2019) and predominantly male people (Moradbakhti et al., 2022). Further, age and gender were also found to moderate the intention to use service chatbots (Jyothsna et al., 2024). Thus, we expected that participants’ intention to use social chatbots is higher in younger people (H13) and males (H14).

### 1.2 The role of anthropomorphism for potential users of social chatbots

The second aim of this study is to scrutinize whether a human-like appearance of social chatbots affects the usage intention of potential users (RQ2). Correlative findings suggest that anthropomorphism is positively related to usage intention of service chatbots (Blut et al., 2021; Ling et al., 2021; Yanxia et al., 2024). Further, experimental studies found that higher anthropomorphism resulted in better interaction experience (Rhim et al., 2022) and that the manipulation of the name and facial appearance of chatbots influences their human-like perception (Seeger et al., 2021). However, the effects of anthropomorphism on usage intention in the context of social chatbots are largely unclear. Here, we expected that social chatbots with a more human-like appearance regarding their name and avatar yield higher usage intention than chatbots without a human-like name and avatar (H15).

## 2 Materials and methods

### 2.1 Participants

Sample size was determined for the linear regression model with 14 independent variables, based on a test power of 0.80, a significance level of 0.05, and a medium-sized effect of *f*^2^ = 0.15. The medium-sized effect was expected due to the number of theory-based independent variables in our model, and since previous studies with similar independent variables have found large effects (e.g., Jyothsna et al., 2024; Wang et al., 2024). The minimum sample size was *n* = 135, and 192 participants completed the study with informed consent. Overall, we excluded 12 participants: one participant stated to be under 18 years of age; nine participants responded to a self-report item at the end of the study that they did not carefully complete the study; two participants showed too long study completion times of more than 1 h based on the 99th percentile (no participant showed study completion times below the first percentile). We analyzed the data of 180 participants (129 female, 49 male, 2 diverse) aged 18–82 years (*M* = 28.68; *SD* = 13.34). Participants were recruited via the social media platforms Facebook, Reddit, and WhatsApp with a focus on groups about social chatbots and research studies. Only psychology students were compensated for their participation in terms of 0.5 subject hours (for a detailed overview of sample characteristics, see Supplementary Table 1, and for a detailed overview of participants’ prior experience with chatbots, see Supplementary Table 2).

### 2.2 Design and procedure

**FIGURE 2:**
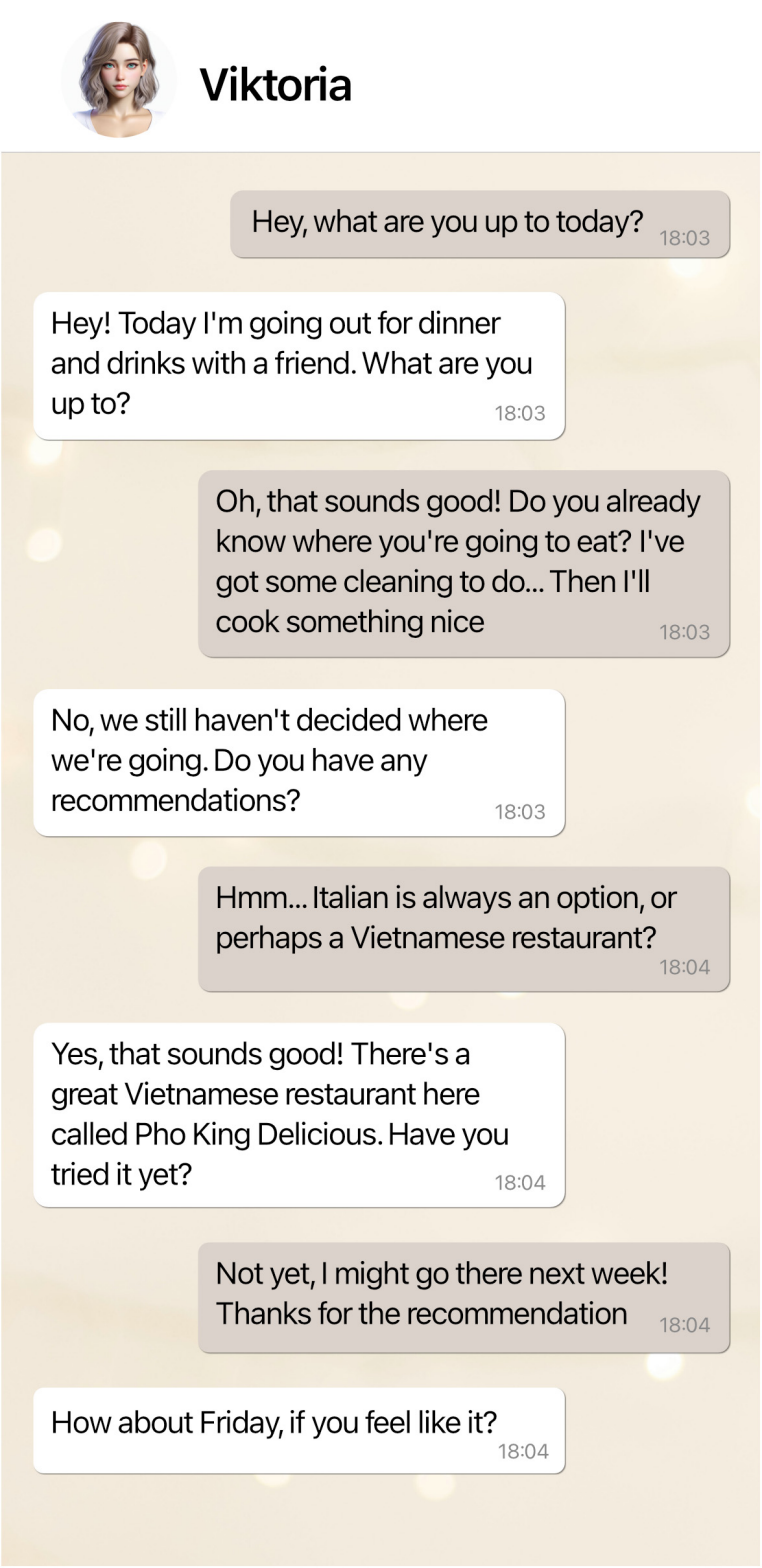
Example conversation between a social chatbot (text on the left side) and a human user (text on the right side).

**FIGURE 3:**
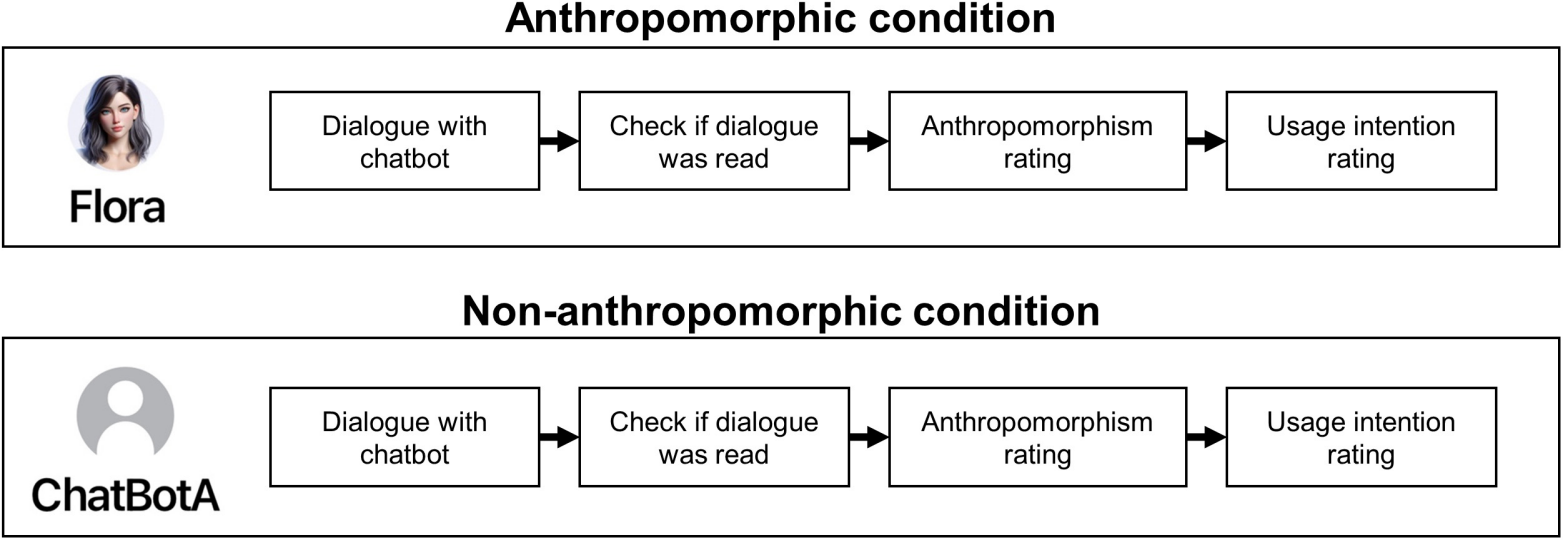
Procedure of the experimental part on the role of anthropomorphism in the intention to use social chatbots.

Our study consisted of a correlational part followed by a within-subjects experimental part. First, participants read a definition describing social chatbots as “intelligent dialogue systems that are able to hold conversations with humans, e.g., about their hobbies, daily life, and emotions.” After providing informed consent, participants completed the correlational part that started with a prototypical dialogue between a social chatbot and a human user (see Figure 2). Participants were asked to carefully read the chat and had to confirm to have read it. Based on the prototypical dialogue, participants rated the anthropomorphism of the social chatbot and their intention to use it. The example dialogue was followed by items on perceived usefulness, perceived ease of use, attitude, perceived behavioral control, social reputation, and subjective norm. Then, participants rated their disposition to trust technology and their disposition to trust people, followed by their experience with chatbots, attachment anxiety, and social support. Between the correlational and experimental part, we collected information on participants’ age, gender, education, residence, and job as well as more detailed information on their prior experience with chatbots (preferred chatbot app, chatbot’s gender, relationship mode, usage behavior, and how they became aware of chatbots). Then, the experimental part started, and participants were instructed to carefully read five chats with social chatbots, and to rate the anthropomorphism of each social chatbot and their intention to use it. Again, participants had to indicate to have read each chat (see Figure 3).

### 2.3 Materials

All conversations with the social chatbots were fictional and created with ChatGPT (version 3.5) based on available chats with chatbots from Brandtzaeg and Følstad (2017). For the experimental part, we created 20 chats of comparable length and eventually selected the five chats with the most comprehensible and natural language. For the anthropomorphism condition, we designed comic-like avatars as in popular social chatbot apps such as Replika. Details on the stimulus creation and the final stimuli can be found in the Supplementary material.

### 2.4 Measures

We adapted measures from validated scales or formulated own items based on previous research and recommendations. All items were rated on a seven-point scale from 1 (*completely disagree*) to 7 (*completely agree*).

#### 2.4.1 Usage intention

The measure for usage intention was adapted from Venkatesh and Bala (2008) and translated into German using translation-back translation (Brislin, 1970). Regarding the prototypical dialogue with the chatbot, usage intention was assessed based on four items (e.g., “I plan to use social chatbots for conversations in the near future”) with very good internal consistency (Cronbach’s α = 0.94). Internal consistency was also good regarding the stimuli in the anthropomorphic condition (all α ≥ 0.78) and in the non-anthropomorphic condition (all α ≥ 0.76).

#### 2.4.2 Technology acceptance measures

Measures for perceived usefulness and perceived ease of use, social reputation, and subjective norm were also adapted from Venkatesh and Bala (2008) and translated into German using translation-back translation. We used four items for perceived usefulness (e.g., “I think using social chatbots can help me to have conversations”) (α = 0.85), four items for perceived ease of use (e.g., “I think the use of social chatbots is clear and understandable”) (α = 0.72), three items for social reputation (e.g., “People in my social circle who use social chatbots for conversations have more prestige than those who do not use social chatbots”) (α = 0.87), and three items for subjective norm (e.g., “People who are influential in my behavior think I should use social chatbots for conversations”) (α = 0.76). Experience with social chatbots was measured via five items adapted from Rüth et al. (2022) (e.g., original: “I would describe myself as a gamer”; adapted: “I would describe myself as a user of social chatbots”), resulting in very good internal consistency (α = 0.96).

#### 2.4.3 Attitude and perceived behavioral control

Based on sample items and recommendations from Ajzen (2020), we formulated three items each for attitude (e.g., “Having conversations with social chatbots is good”) and perceived behavioral control (e.g., “I am convinced that I can use social chatbots for conversations”). Internal consistency was good for attitude (α = 0.84) yet inacceptable for perceived behavioral control (α = 0.44). Item deletion would not have resulted in acceptable internal consistency, so that the items for perceived behavioral control were not combined to a scale. Only the first item showed a significant bivariate correlation with usage intention and was therefore included in the regression model.

#### 2.4.4 Anthropomorphism

Anthropomorphism was assessed by using a seven-level semantic differential by Bartneck et al. (2009), which has already been used in the context of social chatbots (Pentina et al., 2023). The instrument was translated into German using translation-back translation and consists of the following four opposing word pairs: (1) machine-like vs. human-like, (2) artificial vs. lifelike, (3) communicates unnaturally vs. communicates naturally, and (4) has no will of its own vs. has a will of its own. Internal consistency was good regarding the prototypical dialogue with the chatbot (α = 0.77), regarding the stimuli in the anthropomorphic condition (all α ≥ 0.88), and regarding the stimuli in the non-anthropomorphic condition (all α ≥ 0.89).

#### 2.4.5 Attachment anxiety

Attachment anxiety was measured with the anxiety subscale of the experiences in close relationships-revised questionnaire from Ehrenthal et al. (2021). We adapted all four items to refer to close reference persons instead of romantic partners (e.g., original: “I often worry that my partner will not want to stay with me”; adapted: “I often worry that close reference persons will not want to stay with me”), with good internal consistency (α = 0.88).

#### 2.4.6 Social support

Social support was assessed using the brief form of the perceived social support questionnaire from Lin et al. (2018). The scale contains six items (e.g., “I experience a lot of understanding and security from others”) and resulted in good internal consistency (α = 0.90).

#### 2.4.7 Disposition to trust technology and people

To measure disposition to trust technology, we translated all three items for disposition to trust from Delgosha and Hajiheydari (2021) into German using translation-back translation (e.g., “I generally give a technology the benefit of the doubt when I first use it”) (α = 0.90). To measure disposition to trust people, we translated the propensity to trust scale from Frazier et al. (2013) into German using translation-back translation, which consists of four items (e.g., “I usually trust people until they give me a reason not to trust them”) (α = 0.88).

### 2.5 Data analysis

To examine the expected relationships between participants’ personal characteristics and their intention to use social chatbots (H1-H14), we calculated bivariate correlations. Further, we calculated a linear regression model with participants’ intention to use social chatbots as dependent variable and their personal characteristics as independent variables. We checked relevant statistical assumptions for multiple regression analysis (cf. Poole and O‘Farrell, 1971): linearity, normality, and homoscedasticity were given, and there was independence of observations (Durbin-Watson statistic = 1.98) and no multicollinearity (VIF ≤ 3.08). Further, we used bootstrapping for inferential tests as suggested (cf. Hayes and Cai, 2007). We exploratively calculated separate regression models to provide insights on the contribution of each underlying framework and construct set (cf. Figure 1). Correlational analyses were based on data of female and male participants (*n* = 178) since the subgroup of two diverse participants in our sample was too small for gender-related analyses (H14).

To evaluate whether differences in anthropomorphism of social chatbots result in a different intention to use social chatbots (RQ2), we conducted a 5 (social chatbots) × 2 (anthropomorphism vs. non-anthropomorphism condition) repeated measures ANOVA with intention to use social chatbots as dependent variable. We also added age and gender as covariates to check their potential moderating role and the robustness of the results.

## 3 Results

Descriptive statistics for all variables are shown in Supplementary Table 3. One-sample *t*-tests showed high levels of perceived ease of use, perceived behavioral control, disposition to trust people, and social support (all *p*s < 0.001). In contrast, levels were low for perceived usefulness, attitude towards social chatbots, social reputation, subjective norm, experience with social chatbots, attachment anxiety, and the intention to use the prototypical social chatbot (all *p*s < 0.001). The disposition to trust technology (*p* = 0.485) and anthropomorphism of the prototypical social chatbot (*p* = 0.908) were not different from the scale’s midpoint.

### 3.1 Personal characteristics and usage intention (RQ1)

As shown in Table 1, we found large bivariate correlations between participants’ intention to use social chatbots and perceived usefulness (*r* = 0.64), subjective norm (*r* = 0.52), experience with social chatbots (*r* = 0.84), attitude towards social chatbots (*r* = 0.66), perceived behavioral control (*r* = 0.47), and social support (*r* = –0.51). Bivariate correlations were moderate-to-large for social reputation (*r* = 0.29), disposition to trust technology (*r* = 0.15), disposition to trust people (*r* = 0.17), and gender (*r* = –0.20). However, correlations were not significant between participants’ intention to use social chatbots and perceived ease of use (*r* = –0.04), anthropomorphism (*r* = 0.07), attachment anxiety (*r* = 0.14), and age (*r* = 0.11). More detailed information on regression coefficients and confidence intervals can be found in Supplementary Table 4.

**TABLE 1:** The role of personal characteristics in participants’ intention to use social chatbots.

	Intention to use social chatbots (*R*^2^ = 0.75, *F* = 34.85, *p* < 0.001)		
Independent variables	*r*	β	*p*
Age	0.11	0.08	0.088
Gender (0 = male, 1 = female)	−0.20**	0.03	0.522
Perceived usefulness	0.64***	0.10	0.111
Perceived ease of use	−0.04	−0.06	0.204
Social reputation	0.29***	−0.01	0.914
Subjective norm	0.52***	0.05	0.467
Experience with social chatbots	0.84***	0.58	<0.001
Attitude towards social chatbots	0.66***	0.18	0.009
Perceived behavioral control (single item)^a^	0.47***	0.03	0.521
Anthropomorphism	0.07	−0.01	0.705
Attachment anxiety	0.14	0.01	0.758
Social support	−0.51***	−0.10	0.068
Disposition to trust technology	0.15*^b^	−0.06	0.199
Disposition to trust people	−0.17*^b^	0.03	0.518

*r* = bivariate correlation between independent and dependent variable; β = standardized regression coefficients of multiple regression analysis and associated *p*-value (bootstrapped via 10,000 iterations, two-tailed), *p < 0.05, **p < 0.01, ***p < 0.001.

*^a^*Due to low internal consistency, the three items on perceived behavioral control were not summarized into a scale. Only the first item had a significant bivariate correlation with usage intention and was therefore included in the regression model.

*^b^*Not significant considering multiple comparisons (Holm correction).

According to the multiple regression model, all independent variables could explain 74.96% of the variance in the intention to use social chatbots. Particularly, experience with social chatbots and attitude towards social chatbots showed a significant positive relation to the intention to use social chatbots. Thus, only two out of the 14 considered independent variables showed a significant correlation in the full regression model. Exploratory regression model comparisons indicate that age and gender alone can explain a small yet significant amount of variance, which significantly increases after adding technology acceptance measures, after adding theory of planned behavior measures, but not after adding the remaining empirical measures (see Supplementary Table 5). Further, results did not change when using the full perceived behavioral control scale instead of the single item in the regression model.

### 3.2 Anthropomorphism and usage intention (RQ2)

Anthropomorphism ratings were descriptively higher for social chatbots with human-like appearances and names (*M* = 3.62; *SD* = 1.31) compared to social chatbots with generic appearances and names (*M* = 3.56; *SD* = 1.32), yet this difference was not significant and rather small [*t*(179) = 1.32; *p* = 0.190; *d* = 0.10]. We evaluated the expected differences in usage intention based on a 5 (social chatbots) × 2 (anthropomorphism vs. non-anthropomorphism condition) repeated measures ANOVA with usage intention as dependent variable (Greenhouse-Geisser correction applied). We found a main effect of social chatbots, *F*(3.00, 537.34) = 16.34, *p* < 0.001, η_p_^2^ = 0.08, and the expected main effect of anthropomorphism, *F*(1.00, 179.00) = 7.85, *p* = 0.006, η_p_^2^ = 0.04. There was no interaction between social chatbots and anthropomorphism, *F*(3.77, 675.39) = 1.16, *p* = 0.329, η_p_^2^ = 0.01. Based on pairwise comparisons (Bonferroni-corrected), the last chatbot received higher usage intention ratings than all other chatbots (all *p*s < 0.001). When age and gender were added as covariates, there was no significant main effect of anthropomorphism (*p* = 0.072), but also no interaction between anthropomorphism and age (*p* = 0.917) or gender (*p* = 0.115). Anthropomorphism and usage intention ratings of all five chatbots in the anthropomorphic condition and non-anthropomorphic condition can be found in Supplementary Figure 1.

## 4 Discussion

Much research has focused on transactional and functional chatbots such as service chatbots in commercial contexts. In contrast, social chatbots may be valuable tools to accompany and support people in their daily life. We investigated the acceptance of people who are mostly unexperienced with social chatbots regarding key constructs according to theoretical and empirical considerations, including the role of anthropomorphism in terms of a more human-like appearance. The correlational part of this study shows that the intention to use social chatbots is mainly related to experience and attitude regarding social chatbots, followed by perceived usefulness and social support. Notably, a relatively sparse model including sociodemographic variables and technology acceptance measures explained most of the variance in usage intention, which only slightly increased when theory of planned behavior measures and empirically grounded measures were also considered. Further, the bivariate correlations indicate that the intention to use social chatbots of potential users increases with their perception of social chatbots as controllable, trustworthy, and more regarded and recommended by others. Potential users are also more likely to use social chatbots when they are male, have less trust in other people, and receive less social support. In contrast, intention was not related to age, perceived ease of use, attachment anxiety, and anthropomorphism. However, the experimental part of this study indicates that anthropomorphism may still play a role in potential users’ intention to use social chatbots. More specifically, we found that social chatbots with female avatars and popular names received higher usage intention ratings compared to chatbots with schematic avatars and neutral names. Still, anthropomorphism ratings were only slightly higher for social chatbots with a human-like appearance and name compared to a generic appearance and name, suggesting a rather weak role of such general aesthetic and demographic anthropomorphic cues on anthropomorphism. Taken together, we identified relevant constructs for the intention to use social chatbots, whereas a human-like appearance seems to be of lower relative importance but nevertheless can co-determine the intention to use social chatbots.

Further, participants in our study reported a low intention to use social chatbots along with low perceived usefulness, attitude, social reputation, and subjective norm regarding their use. Still, perceived ease of use and perceived behavioral control were high. Also, participants reported moderate disposition to trust technology but high disposition to trust people, low attachment anxiety, and high social support. Thus, there may be lower interest in using social chatbots among people with such personal characteristics.

### 4.1 Limitations and outlook

Our findings are related to some limitations offering several avenues for future research. First, the regression model explained a substantial amount of variance, yet the unexplained variance could be unraveled by considering additional variables. While we have considered several theoretical approaches, there also are extended versions of the technology acceptance model (e.g., Liu et al., 2024) and related theories (e.g., Balakrishnan et al., 2022) as well as further theories related to chatbots (Bialkova, 2024; Chaturvedi et al., 2023). Moreover, the role of the theory of planned behavior and perceived behavioral control in specific could be further explored since the corresponding instrument showed a low internal consistency.

Second, we used the same prompt to create female-like avatars and chats about daily life for our social chatbots, who all had popular names. Still, one chatbot received higher ratings for usage intention and anthropomorphism, so that not all stimuli in one condition were perceived similarly. This chatbot provided task-oriented social support for handling a house plant, illustrating that social chatbots can offer social interaction but also task-related social support. To investigate differences in the perception of chatbot stimuli, anthropomorphism ratings could also be related to eye-tracking parameters determining the visual attention towards the avatars and names of social chatbots and towards the chat content. Further, anthropomorphic features may play a different role in real-time interactions with social chatbots or when social chatbots are animated or visible across the screen. In this regard, the visual representation of the chatbot in our stimuli was of a size comparable to messenger apps, which yet seems be a rather subtle anthropomorphic cue based on the small difference we found in the anthropomorphism ratings. Thus, future research on usage intention could investigate the relative importance of aesthetic and demographic anthropomorphic cues, for instance, by comparing effects of aesthetic, interactional, and functional anthropomorphic cues (e.g., Xin and Liu, 2025; Zhang et al., 2025).

Third, our findings are based on quantitative responses of mostly psychology students and our sample overall may be subject to self-selection bias. So, future research on the usage intention of other potential user groups using various sampling strategies is needed. In specific, studies on frequent or intense users of social chatbots could provide important complementary evidence and allow to further unravel the meaning of usefulness, social support, and other key variables identified in this study.

## Data Availability

The raw data supporting the conclusions of this article will be made available by the authors, without undue reservation.
