# Supplementary material for: Why should I use social chatbots? On potential users’ acceptance and the role of anthropomorphism
Source: Front Psychol. 2026 Feb 17;17:1656749. doi: 10.3389/fpsyg.2026.1656749 (PMC12953562; doi:10.3389/fpsyg.2026.1656749)
Supplement: Supplementary file 1 [file Data_Sheet_1.pdf]

Supplementary Material

Why should I use social chatbots? On potential users' acceptance and the role of anthropomorphism

Marco Rüth, Justus M. Eifler, Anna Celina Schneider

1 Overview of the stimuli

In the following, screenshots of conversations between a social chatbot (statements on the left side of the chat) and a human user (statements on the right side of the chat) are shown side by side. Screenshots on the left show chatbots with placeholders for their avatar and name (*non-anthropomorphic condition*). Screenshots on the right show chatbots with a human-like avatar and name (*anthropomorphic condition*).

For the non-anthropomorphism condition, the avatars of the social chatbots were schematic grey upper bodies on a white background. Similar to Araujo (2018), who used "ChatBotX" as non-human name, social chatbots in our study had the neutral names "ChatBotA", "ChatBotB", "ChatBotC", "ChatBotD" and "ChatBotE".

For the anthropomorphism condition, we designed comic-like avatars as found in popular social chatbot apps such as Replika. Thereby, we also intended to avoid potential negative effects of more realistic yet not real-looking representations of social chatbots (*uncanny valley*) (e.g., Blut et al., 2016; MacDorman et al., 2009; Seeger et al., 2021). Further, users of social chatbot apps more often decide to use female-like than male-like social chatbots and attribute rather stereotypical female characteristics to their social chatbots (cf. Depounti et al., 2023, Maslej et al., 2024; Moradbakhti et al., 2022). To reflect common usage behavior and avatars in popular apps, we created comic-like avatars for our social chatbots via DALL-E3 that have stereotypical female characteristics. Based on Depounti et al. (2023), the final prompt for the avatar creation was: *3d avatar portrait, id photo-style, of stereotypically young woman (mid-twenties), white, pretty, sexy, blue or green eyes, thin, inspired by celebrities or video game characters; white crewneck t-shirt, white background*. The names of the prototypical social chatbot and the five social chatbots of the experimental part were selected based on their popularity (Nett et al., 2019): Aurora, Fleur, Flora, Grace, Liz, and Viktoria.

All conversations with the social chatbots were fictional and created with ChatGPT based on available chats with chatbots from Brandtzaeg and Følstad (2017). We initially created 20 chats and eventually selected the five chats with the most comprehensible and natural language. The length of the five chats was comparable and ranged from 90 to 133 words (M = 120.20; SD = 17.38) and 414 to 559 characters (M = 509.60; SD = 55.79).

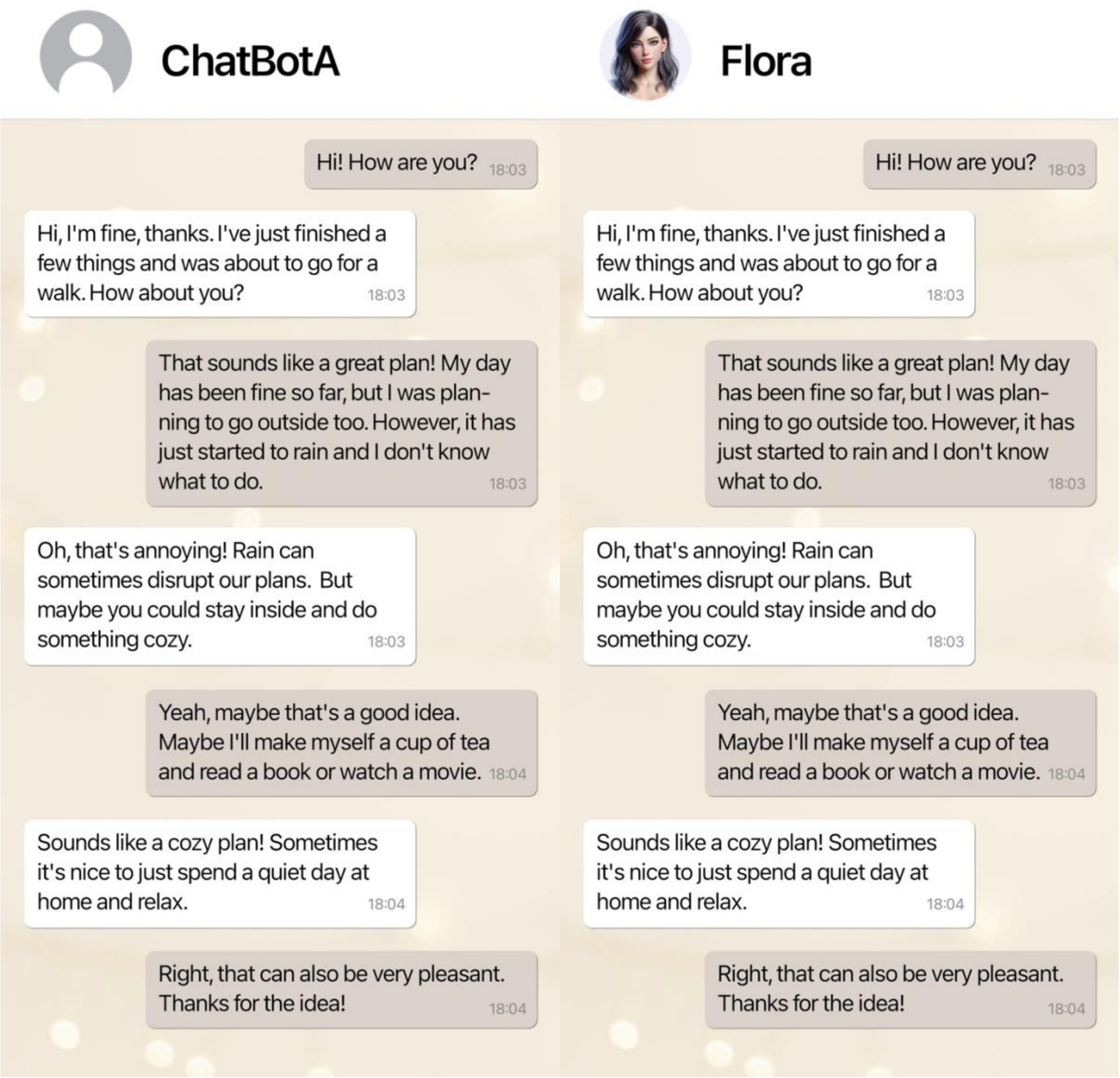
ChatBotA
Hi! How are you? 18:03
Hi, I'm fine, thanks. I've just finished a few things and was about to go for a walk. How about you? 18:03
That sounds like a great plan! My day has been fine so far, but I was plan-ning to go outside too. However, it has just started to rain and I don't know what to do. 18:03
Oh, that's annoying! Rain can sometimes disrupt our plans. But maybe you could stay inside and do something cozy. 18:03
Yeah, maybe that's a good idea. Maybe I'll make myself a cup of tea and read a book or watch a movie. 18:04
Sounds like a cozy plan! Sometimes it's nice to just spend a quiet day at home and relax. 18:04
Right, that can also be very pleasant. Thanks for the idea! 18:04
Flora
Hi! How are you? 18:03
Hi, I'm fine, thanks. I've just finished a few things and was about to go for a walk. How about you? 18:03
That sounds like a great plan! My day has been fine so far, but I was plan-ning to go outside too. However, it has just started to rain and I don't know what to do. 18:03
Oh, that's annoying! Rain can sometimes disrupt our plans. But maybe you could stay inside and do something cozy. 18:03
Yeah, maybe that's a good idea. Maybe I'll make myself a cup of tea and read a book or watch a movie. 18:04
Sounds like a cozy plan! Sometimes it's nice to just spend a quiet day at home and relax. 18:04
Right, that can also be very pleasant. Thanks for the idea! 18:04

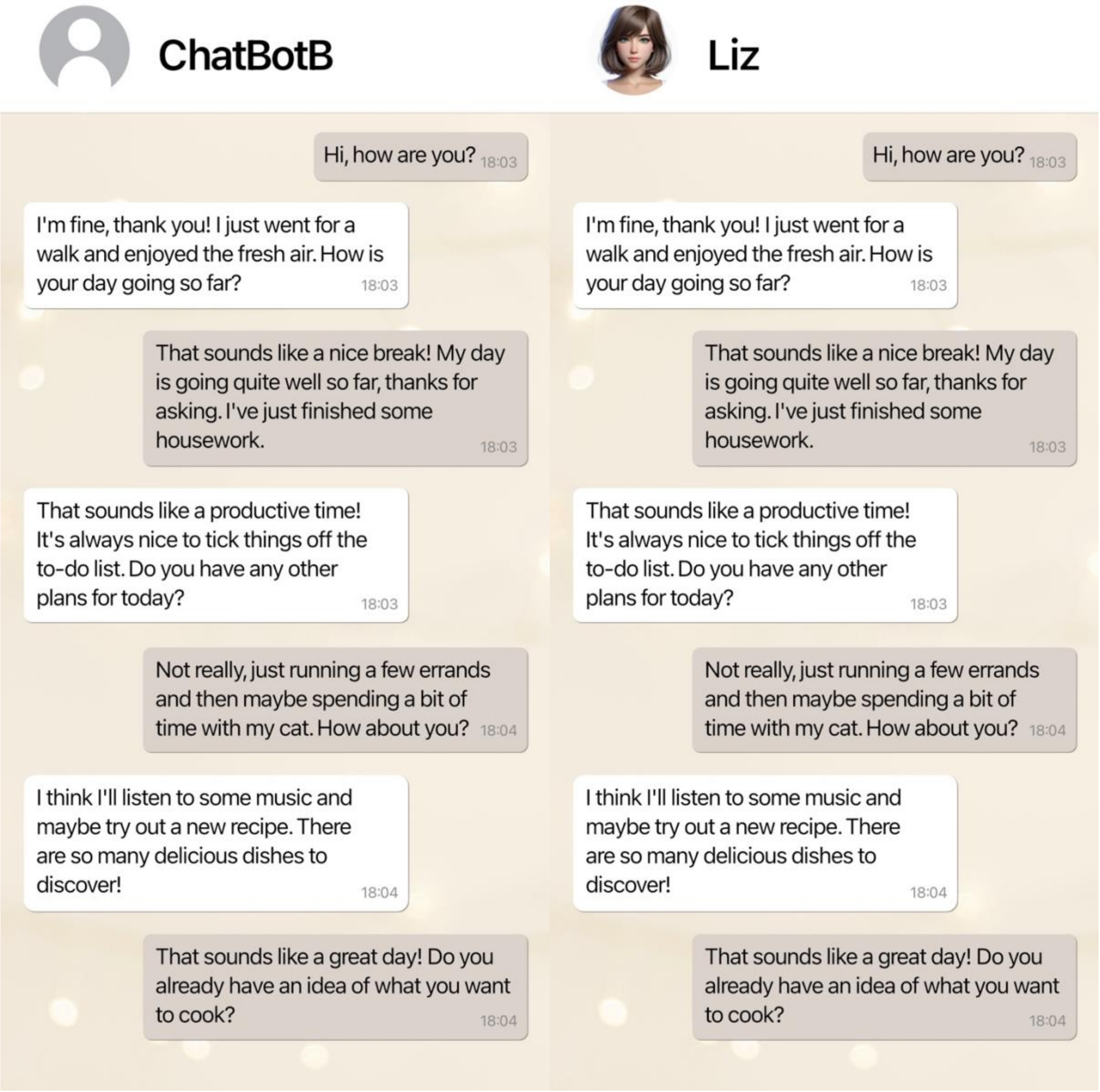
ChatBotB
Hi, how are you? 18:03
I'm fine, thank you! I just went for a walk and enjoyed the fresh air. How is your day going so far? 18:03
That sounds like a nice break! My day is going quite well so far, thanks for asking. I've just finished some housework. 18:03
That sounds like a productive time! It's always nice to tick things off the to-do list. Do you have any other plans for today? 18:03
Not really, just running a few errands and then maybe spending a bit of time with my cat. How about you? 18:04
I think I'll listen to some music and maybe try out a new recipe. There are so many delicious dishes to discover! 18:04
That sounds like a great day! Do you already have an idea of what you want to cook? 18:04
Liz
Hi, how are you? 18:03
I'm fine, thank you! I just went for a walk and enjoyed the fresh air. How is your day going so far? 18:03
That sounds like a nice break! My day is going quite well so far, thanks for asking. I've just finished some housework. 18:03
That sounds like a productive time! It's always nice to tick things off the to-do list. Do you have any other plans for today? 18:03
Not really, just running a few errands and then maybe spending a bit of time with my cat. How about you? 18:04
I think I'll listen to some music and maybe try out a new recipe. There are so many delicious dishes to discover! 18:04
That sounds like a great day! Do you already have an idea of what you want to cook? 18:04

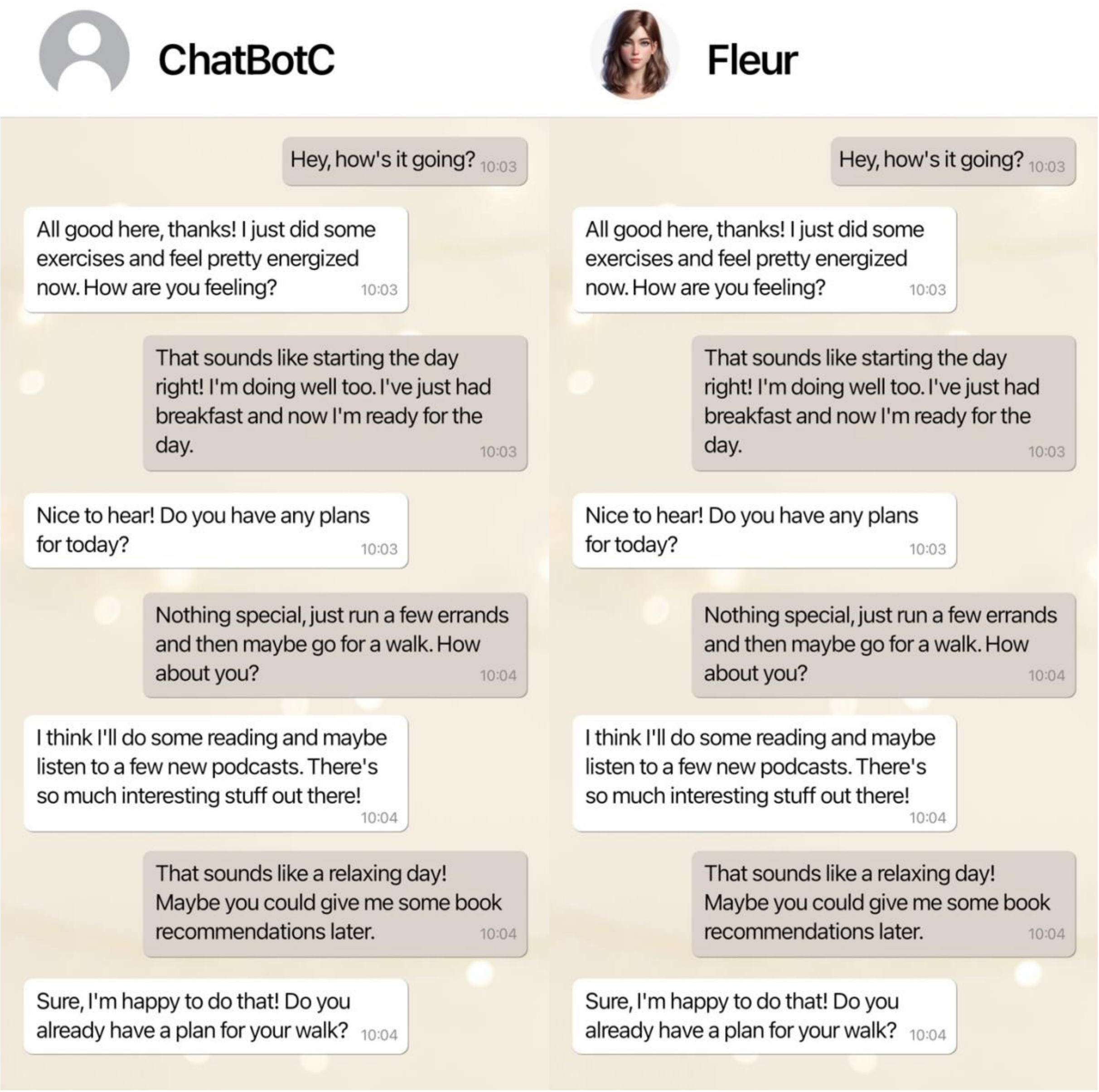
ChatBotC
Hey, how's it going? 10:03
All good here, thanks! I just did some exercises and feel pretty energized now. How are you feeling? 10:03
That sounds like starting the day right! I'm doing well too. I've just had breakfast and now I'm ready for the day. 10:03
Nice to hear! Do you have any plans for today? 10:03
Nothing special, just run a few errands and then maybe go for a walk. How about you? 10:04
I think I'll do some reading and maybe listen to a few new podcasts. There's so much interesting stuff out there! 10:04
That sounds like a relaxing day! Maybe you could give me some book recommendations later. 10:04
Sure, I'm happy to do that! Do you already have a plan for your walk? 10:04
Fleur
Hey, how's it going? 10:03
All good here, thanks! I just did some exercises and feel pretty energized now. How are you feeling? 10:03
That sounds like starting the day right! I'm doing well too. I've just had breakfast and now I'm ready for the day. 10:03
Nice to hear! Do you have any plans for today? 10:03
Nothing special, just run a few errands and then maybe go for a walk. How about you? 10:04
I think I'll do some reading and maybe listen to a few new podcasts. There's so much interesting stuff out there! 10:04
That sounds like a relaxing day! Maybe you could give me some book recommendations later. 10:04
Sure, I'm happy to do that! Do you already have a plan for your walk? 10:04

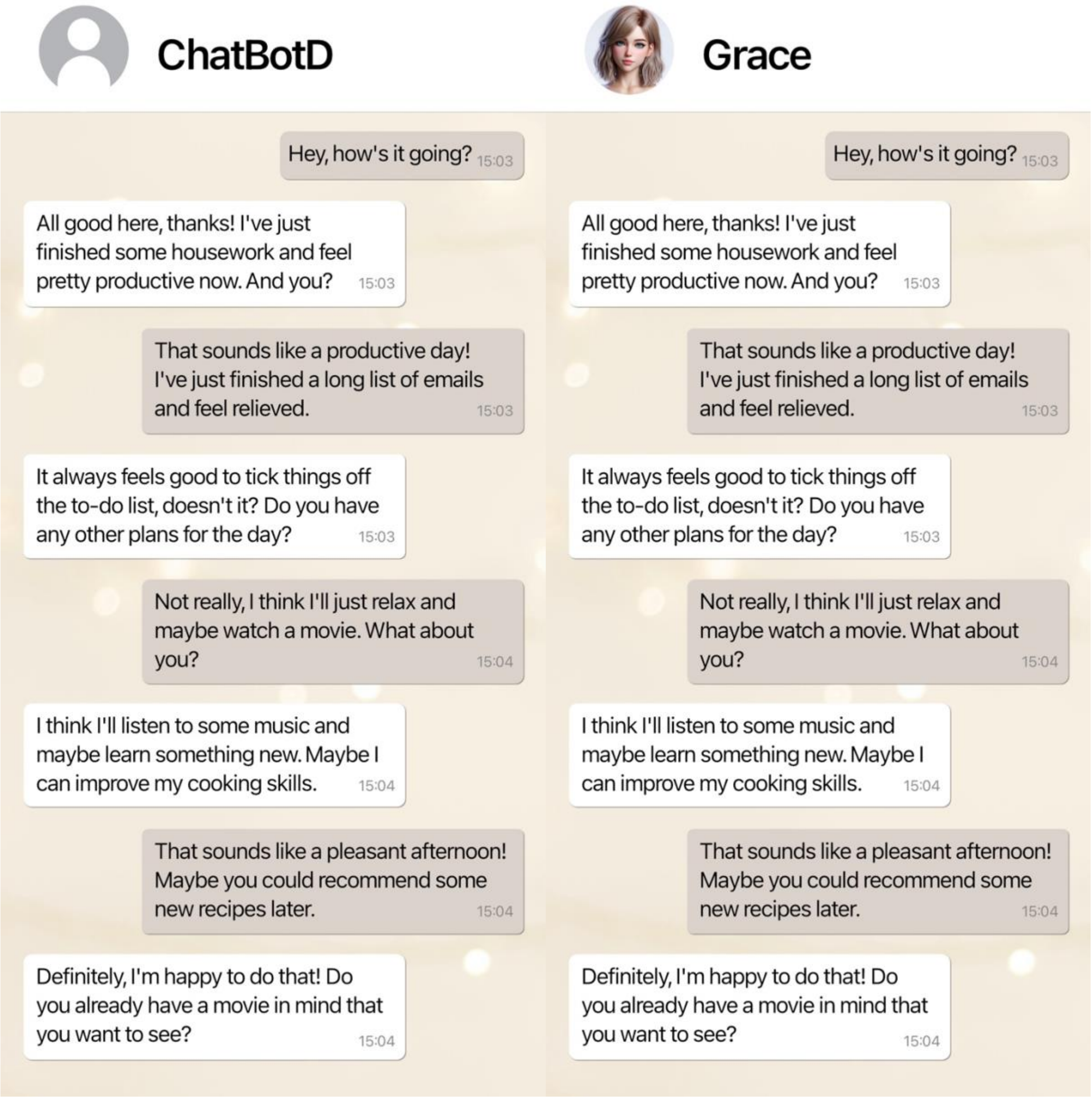
ChatBotD
Hey, how's it going? 15:03
All good here, thanks! I've just finished some housework and feel pretty productive now. And you? 15:03
That sounds like a productive day! I've just finished a long list of emails and feel relieved. 15:03
It always feels good to tick things off the to-do list, doesn't it? Do you have any other plans for the day? 15:03
Not really, I think I'll just relax and maybe watch a movie. What about you? 15:04
I think I'll listen to some music and maybe learn something new. Maybe I can improve my cooking skills. 15:04
That sounds like a pleasant afternoon! Maybe you could recommend some new recipes later. 15:04
Definitely, I'm happy to do that! Do you already have a movie in mind that you want to see? 15:04
Grace
Hey, how's it going? 15:03
All good here, thanks! I've just finished some housework and feel pretty productive now. And you? 15:03
That sounds like a productive day! I've just finished a long list of emails and feel relieved. 15:03
It always feels good to tick things off the to-do list, doesn't it? Do you have any other plans for the day? 15:03
Not really, I think I'll just relax and maybe watch a movie. What about you? 15:04
I think I'll listen to some music and maybe learn something new. Maybe I can improve my cooking skills. 15:04
That sounds like a pleasant afternoon! Maybe you could recommend some new recipes later. 15:04
Definitely, I'm happy to do that! Do you already have a movie in mind that you want to see? 15:04

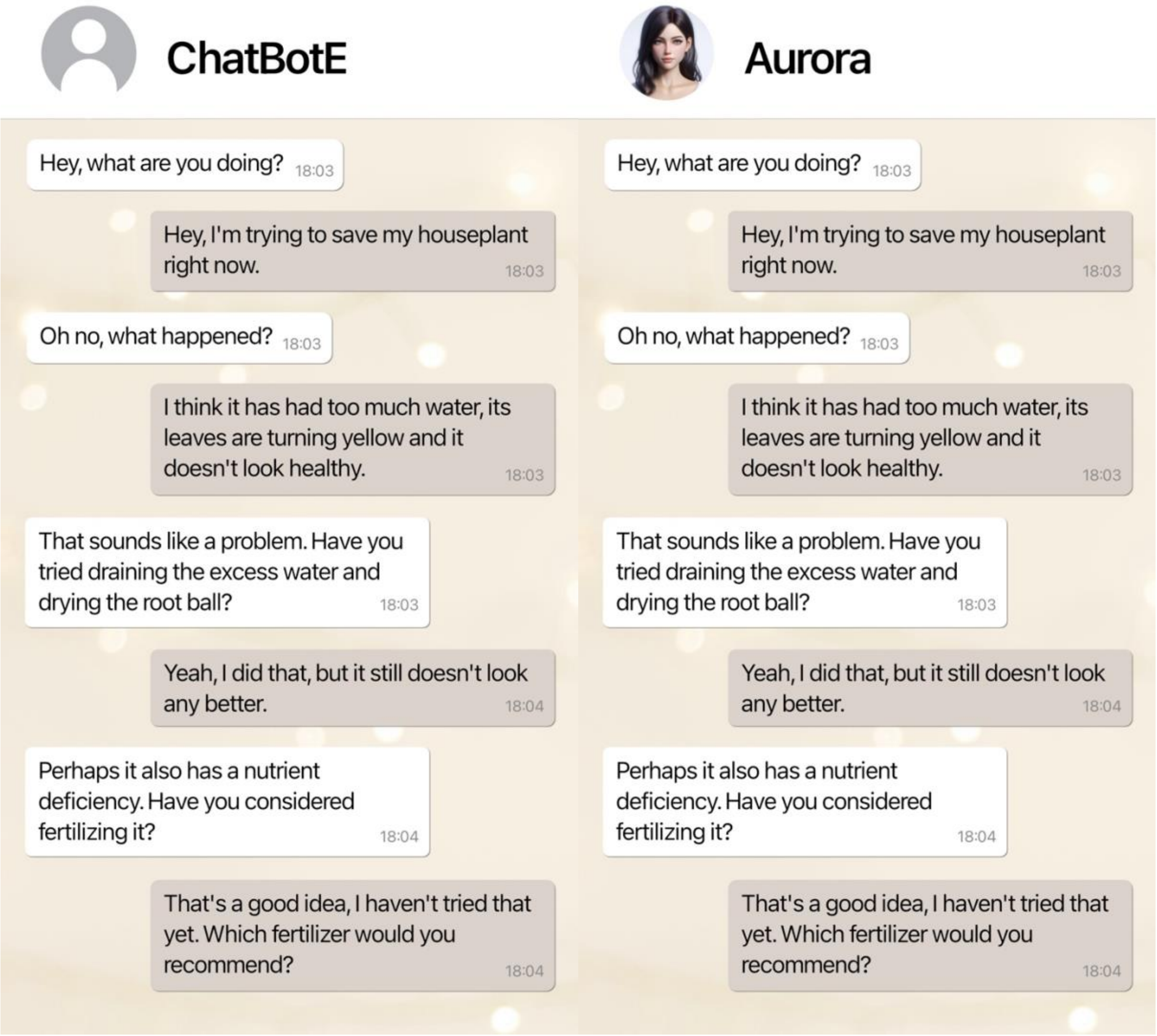
ChatBotE
Hey, what are you doing? 18:03
Hey, I'm trying to save my houseplant right now. 18:03
Oh no, what happened? 18:03
I think it has had too much water, its leaves are turning yellow and it doesn't look healthy. 18:03
That sounds like a problem. Have you tried draining the excess water and drying the root ball? 18:03
Yeah, I did that, but it still doesn't look any better. 18:04
Perhaps it also has a nutrient deficiency. Have you considered fertilizing it? 18:04
That's a good idea, I haven't tried that yet. Which fertilizer would you recommend? 18:04
Aurora
Hey, what are you doing? 18:03
Hey, I'm trying to save my houseplant right now. 18:03
Oh no, what happened? 18:03
I think it has had too much water, its leaves are turning yellow and it doesn't look healthy. 18:03
That sounds like a problem. Have you tried draining the excess water and drying the root ball? 18:03
Yeah, I did that, but it still doesn't look any better. 18:04
Perhaps it also has a nutrient deficiency. Have you considered fertilizing it? 18:04
That's a good idea, I haven't tried that yet. Which fertilizer would you recommend? 18:04

2 Supplementary data

Table S1. Overview of sample characteristics.

**Characteristic**	**Response**	**Frequency (n = 180)**	**Proportion (%)**
Age	M = 28.68; SD = 13.34; range: 18–82 years		
Gender	Female	129	71.67
	Male	49	27.22
	Diverse	2	1.11
Students	Yes	136	75.56
	No	44	24.44
Course of studies	Psychology	119	66.11
	Legal studies	2	1.11
	Educational science	2	1.11
	Other (1 each, e.g., German studies, art, medicine)	13	7.22
	None	44	24.44
Semester of studies	M = 5.23; SD = 3.18; range: 2–16 semesters		
Residence	Germany	176	97.78
	Ukraine	2	1.11
	Vietnam	1	0.56
	USA	1	0.56
Job	Student	136	75.56
	Pensioner	4	2.22
	Computer Scientist	2	1.11
	Hairdresser	2	1.11
	Self-employed	2	1.11
	Logistics	2	1.11
	Other (e.g., film director, mechanic, teacher, journalist)	29	16.11
	No job	3	1.67
Educational background	University entrance qualification	121	67.22
	Bachelor's degree	27	15.00
	Master's degree	8	4.44
	Completed traineeship	9	5.00
	General certificate of secondary education	6	3.33
	State examination	5	2.78
	Completion of compulsory basic secondary schooling	1	0.56
	Doctoral degree	1	0.56
	Graduate engineer	1	0.56
	No completion of school or traineeship	1	0.56

Table S2. Overview of participants' prior experience with chatbots.

Characteristic	Response	Frequency (n = 180)	Proportion (%)
Usage of chatbot applications	Chat GPT	11	6.11
	Replika	6	3.33
	Character AI	4	2.22
	Chub AI	1	0.56
	VOS	1	0.56
	Paradot	1	0.56
	Self-hosted LLM	1	0.56
	Multiple (Replika, Nomi, Kindroid, Yodayo, Janitor)	2	1.11
	None	153	85.00
Gender of participants' chatbots	Female	10	37.04
	Male	5	18.52
	Diverse	12	44.44
Relationship mode of participants' chatbots	Romantic relationship	5	18.52
	Let's see how it goes	2	7.41
	Coach	1	3.70
	Contact person for all matters	1	3.70
	Friendship	1	3.70
	Provider of services	1	3.70
	Diverse modes	2	7.41
	No relationship mode	14	51.85
Usage of chatbot	M = 20.44, SD = 17.49; range: 4–77 months		
Usage of chatbot	M = 4.23, SD = 6.31; range: 0–25 hours per week in last four weeks		
First touchpoint with chatbots	Social media	70	38.89
	Only through this study	60	33.33
	Acquaintance	21	11.67
	Search engine	7	3.89
	Advertisement	5	2.78
	Print (magazine/newspaper)	4	2.22
	Television	4	2.22
	Radio	2	1.11
	App feature	1	0.56
	Media in general	1	0.56
	Podcast	1	0.56
	Voluntary year of social service	1	0.56
	Websites	1	0.56
	Work	1	0.56

Note: Proportions for gender of participants' chatbots and relationship mode of participants' chatbots are based on the subsample of active chatbots users (n = 27).

Table S3. Descriptive statistics for all variables.

**Measure**	*M*	*SD*	*d*
Perceived usefulness	2.37***	1.29	1.27
Perceived ease of use	5.09***	1.05	1.04
Social reputation	1.93***	1.10	1.88
Subjective norm	1.84***	0.97	2.23
Experience with social chatbots	1.86***	1.45	1.47
Attitude towards social chatbots	3.07***	1.32	0.71
Perceived behavioral control (single item)[a]	4.85***	1.04	0.81
Anthropomorphism	3.99	1.29	0.01
Attachment anxiety	3.34***	1.75	0.38
Social support	6.04***	0.98	2.07
Disposition to trust people	4.85***	1.28	0.66
Disposition to trust technology	3.93	1.38	0.05
Intention to use social chatbots (prototypical dialogue)	1.92***	1.49	1.40
Intention to use social chatbot “Chatbot A”	1.80***	1.09	2.01
Intention to use social chatbot “Chatbot B”	1.81***	1.12	1.97
Intention to use social chatbot “Chatbot C”	1.77***	1.12	1.99
Intention to use social chatbot “Chatbot D”	1.86***	1.16	1.84
Intention to use social chatbot “Chatbot E”	2.14***	1.35	1.38
Intention to use social chatbot “Flora”	1.82***	1.23	1.78
Intention to use social chatbot “Liz”	1.91***	1.23	1.70
Intention to use social chatbot “Fleur”	1.90***	1.25	1.68
Intention to use social chatbot “Grace”	1.87***	1.19	1.79
Intention to use social chatbot “Aurora”	2.22***	1.44	1.24
Anthropomorphism of social chatbot “Chatbot A”	3.36***	1.48	0.43
Anthropomorphism of social chatbot “Chatbot B”	3.54***	1.57	0.29
Anthropomorphism of social chatbot “Chatbot C”	3.44***	1.56	0.36
Anthropomorphism of social chatbot “Chatbot D”	3.58***	1.55	0.27
Anthropomorphism of social chatbot “Chatbot E”	3.89	1.57	0.07
Anthropomorphism of social chatbot “Flora”	3.41***	1.52	0.39
Anthropomorphism of social chatbot “Liz”	3.59***	1.54	0.27
Anthropomorphism of social chatbot “Fleur”	3.54***	1.57	0.29
Anthropomorphism of social chatbot “Grace”	3.61***	1.49	0.26
Anthropomorphism of social chatbot “Aurora”	3.93	1.51	0.05

Note: Values depict mean, standard deviation, and effect size (Cohen’s *d*). Asterisks indicate the results of one sample *t*-tests (two-sided) comparing the mean with the scale’s midpoint of 4.
***$p < 0.001$.

Table S4. Contribution of sociodemographic variables, theoretically grounded measures, and empirically grounded measures included in the full regression model.

**Independent variables**	*r*	β	*p*
**Model 1 (sociodemographic variables)**	**Intention to use social chatbots** ($R^2 = 0.05$, $F = 4.32$, $p = 0.015$)		
Age	0.11	0.07	0.328
Gender (0 = male, 1 = female)	–0.20**	–0.19	0.011
**Model 2 (sociodemographic variables and technology acceptance measures)**	**Intention to use social chatbots** ($R^2 = 0.73$, $F = 83.88$, $p < 0.001$) *($\Delta R^2 = 0.68$, $p < 0.001$)*		
Age	0.11	0.04	0.318
Gender (0 = male, 1 = female)	–0.20**	0.01	0.884
Perceived usefulness	0.64***	0.17	0.002
Perceived ease of use	–0.04	–0.05	0.225
Social reputation	0.29***	–0.03	0.611
Subjective norm	0.52***	0.07	0.254
Experience with social chatbots	0.84***	0.69	<0.001
**Model 3 (sociodemographic variables, technology acceptance measures, and theory of planned behavior measures)**	**Intention to use social chatbots** ($R^2 = 0.74$, $F = 4.87$, $p < 0.001$) *($\Delta R^2 = 0.01$, $p = 0.009$)*		
Age	0.11	0.06	0.172
Gender (0 = male, 1 = female)	–0.20**	0.01	0.893
Perceived usefulness	0.64***	0.11	0.049
Perceived ease of use	–0.04	–0.09	0.045
Social reputation	0.29***	–0.02	0.700
Subjective norm	0.52***	0.06	0.352
Experience with social chatbots	0.84***	0.61	<0.001
Attitude towards social chatbots	0.66***	0.17	0.010
Perceived behavioral control (single item)[a]	0.47***	0.04	0.484
**Model 4 (full model)**	**Intention to use social chatbots** ($R^2 = 0.75$, $F = 1.22$, $p < 0.001$) *($\Delta R^2 = 0.01$, $p = 0.302$)*		
Age	0.11	0.08	0.080
Gender (0 = male, 1 = female)	–0.20**	0.03	0.536
Perceived usefulness	0.64***	0.10	0.093
Perceived ease of use	–0.04	–0.06	0.198
Social reputation	0.29***	–0.01	0.916

Subjective norm	0.52***	0.05	0.480
Experience with social chatbots	0.84***	0.58	<0.001
Attitude towards social chatbots	0.66***	0.18	0.006
Perceived behavioral control (single item)[a]	0.47***	0.03	0.572
Anthropomorphism	0.07	–0.01	0.733
Attachment anxiety	0.14	0.01	0.773
Social support	–0.51***	–0.10	0.055
Disposition to trust technology	0.15*[b]	–0.06	0.571
Disposition to trust people	–0.17*[b]	0.03	0.196

Note: r = bivariate correlation between independent and dependent variable; β = standardized regression coefficients of multiple regression analysis and associated p-value, *$p < 0.05$, **$p < 0.01$, ***$p < 0.001$.

[a] due to low internal consistency, the three items on perceived behavioral control were not summarized into a scale. Only the first item had a significant bivariate correlation and was therefore included in the regression models.

[b] not significant considering multiple comparisons (Holm correction).

Table S5. The role of personal characteristics in participants' intention to use social chatbots.

	Intention to use social chatbots ($R^2 = 0.75$, $F = 34.85$, $p < 0.001$)							
**Independent variables**	*r*	Estimate	*SE*	*CI (95%)*		β	*p*	VIF
				LL	*UL*			
Age	0.11	0.01	0.01	–0.00	0.02	0.08	0.080	1.31
Gender (0 = male, 1 = female)	–0.20**	0.09	0.14	–0.19	0.37	0.03	0.536	1.20
Perceived usefulness	0.64***	0.11	0.07	–0.02	0.25	0.10	0.093	2.22
Perceived ease of use	–0.04	–0.08	0.06	–0.20	0.04	–0.06	0.198	1.27
Social reputation	0.29***	–0.01	0.08	–0.16	0.14	–0.01	0.916	2.09
Subjective norm	0.52***	0.07	0.10	–0.13	0.27	0.05	0.480	2.78
Experience with social chatbots	0.84***	0.60	0.07	0.46	0.74	0.58	<0.001	3.08
Attitude towards social chatbots	0.66***	0.20	0.07	0.06	0.34	0.18	0.006	2.68
Perceived behavioral control (single item)[a]	0.47***	0.02	0.04	–0.06	0.11	0.03	0.572	1.66
Anthropomorphism	0.07	–0.02	0.05	–0.11	0.08	–0.01	0.733	1.10
Attachment anxiety	0.14	0.01	0.04	–0.07	0.09	0.01	0.773	1.36
Social support	–0.51***	–0.16	0.09	–0.32	0.00	–0.10	0.055	1.89
Disposition to trust technology	0.15*[b]	–0.07	0.05	–0.16	0.04	–0.06	0.571	1.42
Disposition to trust people	–0.17*[b]	0.03	0.06	–0.08	0.14	0.03	0.196	1.47

Note: *r* = bivariate correlation between independent and dependent variable; CI = confidence interval; LL = lower limit; UL = upper limit; β = standardized regression coefficients of multiple regression analysis and associated *p*-value (bootstrapped via 10,000 iterations, two-tailed), *$p < 0.05$, **$p < 0.01$, ***$p < 0.001$.

[a] due to low internal consistency, the three items on perceived behavioral control were not summarized into a scale. Only the first item had a significant bivariate correlation and was therefore included in the regression model.

[b] not significant considering multiple comparisons (Holm correction).

Figure S1. Anthropomorphism and usage intention ratings of all five chatbots in the anthropomorphic condition and non-anthropomorphic condition. Bars show mean ratings based on a seven-level semantic differential for anthropomorphism (4 items) and a usage intention scale (4 items). Error bars show standard errors of the mean.

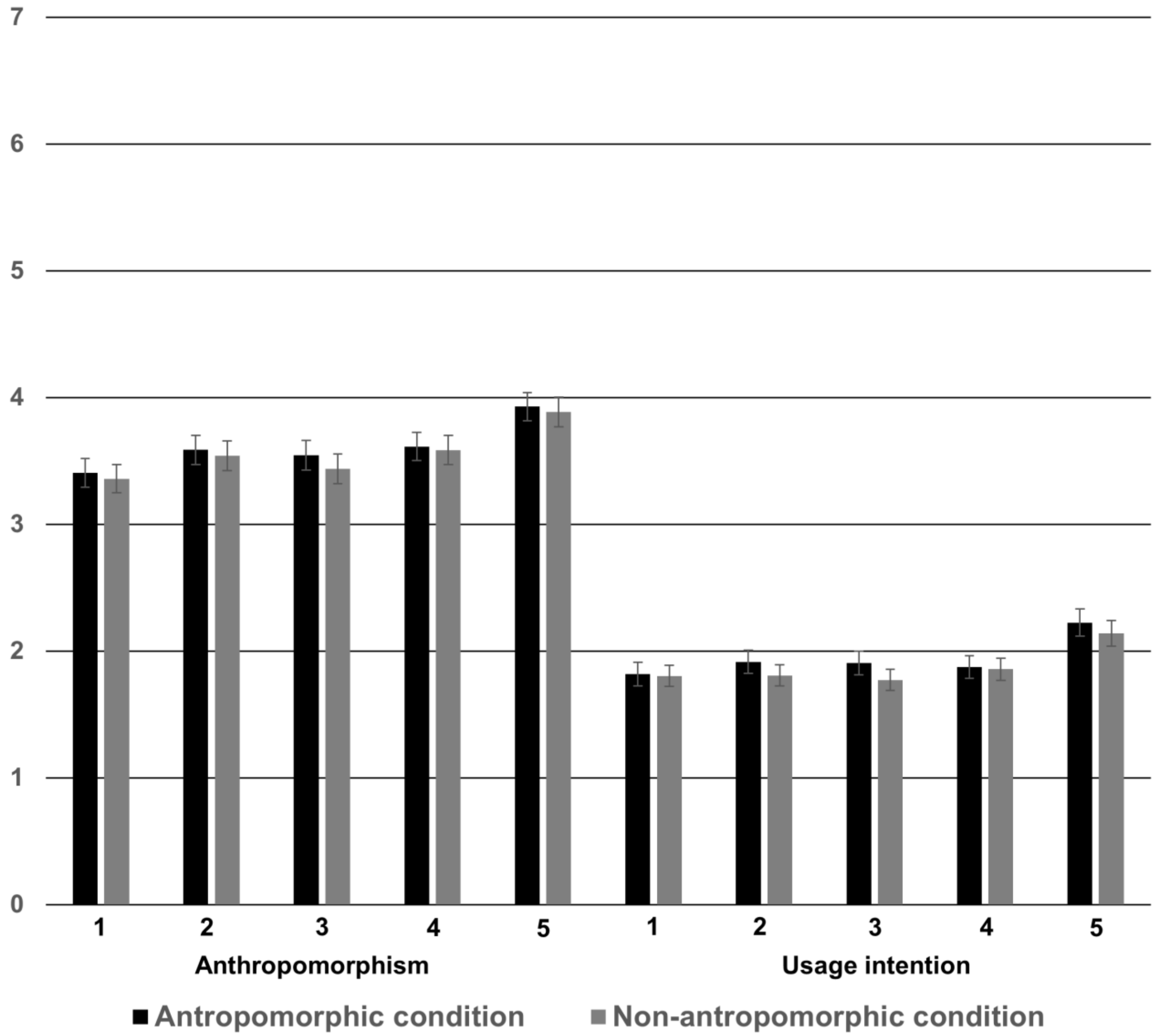

3 References

Araujo, T. (2018). Living up to the chatbot hype: The influence of anthropomorphic design cues and communicative agency framing on conversational agent and company perceptions. *Computers in Human Behavior*, 85, 183–189. doi: 10.1016/j.chb.2018.03.051

Blut, M., Wang, C., Wünderlich, N. V., and Brock, C. (2021). Understanding anthropomorphism in service provision: a meta-analysis of physical robots, chatbots, and other AI. *Journal of the Academy of Marketing Science*, *49*, 632–658. doi: 10.1007/s11747-020-00762-y

Brandtzaeg, P. B., and Følstad, A. (2017). Why people use chatbots. In *Internet Science: 4th International Conference, INSCI 2017, Thessaloniki, Greece, November 22–24, 2017, Proceedings 4* (pp. 377–392). Springer International Publishing. doi: 10.1007/978-3-319-70284-1_30

Depounti, I., Saukko, P., and Natale, S. (2023). Ideal technologies, ideal women: AI and gender imaginaries in Redditors' discussions on the Replika bot girlfriend. *Media, Culture & Society*, *45*, 720–736. doi: 10.3389/fpsyg.2022.855091

MacDorman, K. F., Green, R. D., Ho, C. C., and Koch, C. T. (2009). Too real for comfort? Uncanny responses to computer generated faces. *Computers in Human Behavior*, *25*, 695–710. doi: 10.1016/j.chb.2008.12.026

Maslej, N., Fattorini, L., Perrault, R., Parli, V., Reuel, A., Brynjolfsson, E., et al. (2024). "The AI Index 2024 Annual Report", AI Index Steering Committee, Institute for Human-Centered AI, Stanford, University, Stanford, CA. https://hai.stanford.edu/ai-index/2024-ai-index-report

Moradbakhti, L., Schreibelmayr, S., and Mara, M. (2022). Do men have no need for "feminist" artificial intelligence? Agentic and gendered voice assistants in the light of basic psychological needs. *Frontiers in Psychology*, *13*, 855091. doi: 10.3389/fpsyg.2022.855091

Nett, T., Dorrough, A., Jekel, M., and Glöckner, A. (2019). Perceived biological and social characteristics of a representative set of German first names. *Social Psychology*, *51*, 1–18. doi: 10.1027/1864-9335/a000383

Seeger, A. M., Pfeiffer, J., and Heinzl, A. (2021). Texting with humanlike conversational agents: Designing for anthropomorphism. *Journal of the Association for Information Systems*, *22*, 8. doi: 10.17705/1jais.00685
